# Polymorphism of IFN-γ (+874 T/A) in Syrian patients with chronic hepatitis B 

**Published:** 2017

**Authors:** Mohamad Al Kadi, Fawza Monem

**Affiliations:** 1 *Department of Clinical Biochemistry and Microbiology, Faculty of Pharmacy, Damascus University, Damascus, Syria*; 2 *Clinical Laboratories Department, Al-Assad Hospital, Damascus, Syria*

**Keywords:** IFN-γ, Hepatitis B, Chronic, Polymorphism, Single nucleotide, Syria

## Abstract

**Aim:**

This study aimed to investigate the association of IFN- γ +874 (T/A) polymorphism with susceptibility to chronic HBV infection in the Syrian population.

**Background:**

Accumulating evidence indicate that the inadequate immune responses are responsible for HBV persistency. Therefore, polymorphisms in genes encoding the cytokines, which are responsible for regulation of the immune response, can affect the course and outcome of the infection. The IFN-γ +874 T/A polymorphism affects the expression of IFN-γ, which has been shown to be crucial to HBV clearance.

**Methods:**

In this case-control study, 140 samples were collected (70 healthy individuals, 70 chronic HBV patients), and genomic DNA was isolated. Sequencing and ARMS-PCR were performed to genotype the IFN-γ +874 T/A polymorphism.

**Results:**

Results of this study showed an association between IFN- γ +874 T/A polymorphism and the susceptibility to chronic HBV infection (*P *< 0.05). In addition, results showed that the AA genotype increased the risk of chronicity (OR = 3.05, 95% CI = 1.35 – 6.89), whereas the AT and TT genotypes reduced the risk of chronicity (OR = 0.33, 95% CI = 0.150 – 0.753).

**Conclusion:**

Results of this study conclude that the IFN- γ +874 T/A polymorphism may be associated with the chronic HBV infection, according to the genetic model AA vs. AT&TT.

## Introduction

Chronic hepatitis B (CHB) is a global public health problem, with more than 240 million people chronically infected worldwide, who are at risk for cirrhosis and hepatocellular carcinoma (1). Most adult patients who are infected with HBV recover completely, whereas 5- 10% of them develop a chronic infection (2). A complex combination of environmental, viral and host genetic factors play a role in determining both susceptibility to HBV persistence and the course of infection(3). However, accumulating evidence shows that inadequate immune responses to HBV are responsible for viral persistence (4). Chronic HBV infection elicits very weak T cell responses, while vigorous T cell responses are stimulated at acute, self-limiting HBV infection, with Th1 responses seem to be involved in the clearance of HBV from hepatocytes (5). Therefore, polymorphisms in genes encoding the pro-inflammatory and anti-inflammatory cytokines, which are responsible for regulation of the immune response and Th1/Th2 balance, can affect the course and outcome of the infection (6). Interferon gamma (IFN-γ), the principal Th1 effector cytokine, has shown to be crucial to HBV clearance (7, 8). Many Single Nucleotides Polymorphisms (SNPs) are located in this gene. Nevertheless, only the SNP (+874T/A, rs2430561) is widely studied in this gene (9). This T to A polymorphism coincides with a putative NF-κB binding site, which has functional consequences for the transcription of the human IFN-γ gene (10). Thus, the homozygous T genotype is associated with the ability to produce high levels of IFN-γ, the heterozygous T/A genotype, with intermediate levels, and the homozygous A genotype, with low levels (11). To the best of our knowledge, there is no study available from Syria or Arab countries reporting the effects of these polymorphisms on the outcome of HBV infection. Our study aimed to investigate the association of the Interferon gamma (IFN-γ ) +874T/A (rs2430561) polymorphism with the susceptibility to chronic HBV infection in Syrian patients. 

## Materials and Methods

**Study Subjects**

After the ethics committee approval, written informed consents were obtained. Our case-control study included 140 subjects. Patients were enrolled from the outpatient hepatology clinic at the Damascus Hospital. Controls were healthy blood donors at the Damascus Center for Blood Transfusion. Samples were collected between March 2013 and October 2015.

All patients met the inclusion criteria: (1) positivity of serum HBsAg for more than six months, (2) age more than 20 years, and (3) serum negativity for anti-HCV and anti-HIV. The inclusion criteria for the controls group, healthy individuals, included negativity of serum HBsAg, HBcAg, anti-HCV and anti-HIV.Ages and genders of the control group were matched with the patients group.

**Genomic DNA Extraction**

Peripheral blood samples were collected from study subjects on EDTA sterile tubes for genomic DNA isolation. Genomic DNA was isolated from peripheral white blood cells using Gen^X^TRACT Resin (ViennaLab Diagnostics, Austria) according to manufacturer's instructions. Concentration and purity of DNA samples were checked by NanoDrop. Samples were stored at -20°C.

**Sequencing**

Five PCR reactions were conducted on five randomly chosen DNA samples by GeneAmp® PCR System 9700 (Applied Biosystems®, USA) using: [F]5'-TCGTTGCTCACTGGGATTTTG-3′ and [R] 5′-CATCTACTGTGCCTTCCTGT-3′ (12). The amplified 322 bp products were separated by electrophoresis on a 1.5% agarose gel.

PCR products were purified with High Pure PCR Product Purification Kit (Roche, Switzerland), and sequenced using BigDye® Terminator v3.1 Cycle Sequencing (Applied Biosystems, USA) with the primer 5′-CATCTACTGTGCCTTCCTGT-3′. Capillary electrophoresis was performed with ABI PRISM® 3100-Avant™ Genetic Analyzer® (Applied Biosystems, USA). Consequently, these five samples representing the three different genotypes, were used to optimize and establish the amplification refractory mutation system–polymerase chain reaction (ARMS-PCR) to be used for genotyping the IFN-γ +874 T /A polymorphism.

**The** **amplification refractory mutation system–polymerase chain reaction** **(ARMS-PCR)**

ARMS-PCR method was used to genotype the IFN-γ +874 T/A polymorphism. Two PCRs were conducted for each DNA sample. Each PCR reaction contained the following primers: a forward internal control primer (12), a forward specific primer, and a common reverse primer (10). The common primer served as a reverse primer for the specific and internal control primers (Table 1). Each PCR reaction contained: 10 µL GoTaq® Master mix 2X (Promega, USA), 50 -100 ng of DNA sample, specific primer (2nM), internal control primer (1nM), and reverse primer (2nM); all diluted in free-nuclease water to a final volume of 20 µL. The reactions were performed in GeneAmp® PCR System 9700 (Applied Biosystems, USA) according to the protocol: 95°C for 5 min, followed by 35 cycles of 95°C for 30 sec, 57.5°C for 30 sec and 72°C for 30 sec, then a final extension at 72°C for 5 min. The amplified products were separated on a 1.5% agarose gel containing ethidium bromide with 50 bp DNA ladder (Fermentas, USA), then the gel was imaged and documented by a gel documentation system (Zenith Engineers, India)

**Statistical analysis**

Results were analyzed by SPSS 17.0 software (IBM, USA) and SNPstat http://bioinfo.iconcologia.net/SNPstats_web (13). The quantitative data (ages) were analyzed using student's t-test. The allele frequencies were tested for Hardy-Weinberg equilibrium (HWE) using chi-squared good of fitness test. Logistic regression analysis was used to compare the genotypes between groups, adjusted by age and gender. P-values less than 0.05 were considered significant. Odd ratios (OR) and confidence intervals (95%CI) were calculated in case of significant difference.

## Results

**Characteristics of study subjects**

The mean ages of control subjects and chronic HBV patients were 38.49 ± 11.22 and 40.05 ± 13.16 years, respectively. No significant difference was found between the two groups in term of age, gender, smoking and alcohol status (*P >* 0.05) (Table 2).

**Genotype distribution**

Allele frequencies were in Hardy-Weinberg equilibrium in both groups (*P* > 0.05). 

Comparison of the allele frequencies revealed a significant difference in the distribution of alleles in the two groups (*P <* 0.05) (Table 3). Allele A was higher in the patient group with odd ratio of 1.74 and 95%CI = 1,012 – 3. In terms of the genotype frequency, our results showed a significant difference between the two groups (*P* = 0.021) (Table 4). The AA allele was higher in the patient group 25 (36%) compared to the control group 11 (16%). On the opposite, the AT allele and the allele TT were higher in the control group (41 (58%) and 18 (26%), respectively) compared to the patient group (30 (44%) and 15 (21%), respectively). Occurrence of AA allele increased risk of chronic HBV (*P* = 0.0057, OR = 3.05, 95% CI = 1.35 – 7.52). Whereas, occurrence of AT and TT alleles reduced the risk of chronic HBV with the odd ratio of 0.33 (95% CI = 0.150 – 0.753). 

Testing different genetic models revealed that there was a significant difference between healthy controls and patients for the codominant model (AA vs. AT, AA vs. TT) (*P* < 0.05, OR =3.18, 95%CI =1.35-7.52, OR = 2.78, 95%CI = 1.03 – 7.52, respectively). A significant difference between the two groups was found for the recessive model (AA vs. AT and TT) (*P* < 0.05 OR = 3.05, 95%CI = 1.35 – 6.89) and for the additive model (P <0.05, OR = 1.67, 95%CI = 1.02 – 2.73). On the other hand, there was no significant difference (*P* > 0.05) in the dominant model (TT vs. AA and AT) and over-dominant model (AT vs. AA&TT) (Table 4). According to the Akaike information criterion (AIC), the recessive model (AA vs AT&TT) was the best genetic model, since it has the minimum AIC value (192.2). 

## Discussion

The AA genotype was significantly higher in our patients group compared to controls (36% vs. 16%). This finding was consistent with several studies (11, 14, 15). The AA genotype is correlated with low interferon gamma production (10). Interferon gamma plays an important role in the viral clearance of HBV. Blockade of interferon gamma abolishes the non-cytolytic inhibition of HBV (7) indicating that interferon gamma mediates the control of HBV. This might explain the increased risk of chronic HBV in our patients in correlation with the presence of AA genotype (OR = 3.05, 95% CI = 1.35 – 7.52). On the other hand, many studies did not find an association of the AA genotype with the HBV chronicity (16-19), which might be justified by the high frequency of the AA genotype in their control groups. For instance, high AA genotype frequencies were reported in most Asian ethnic studies (16, 19, 20), as it was 65% in the control group of a Chinese study (19). This suggests that the increased risk of chronic HBV, in term of the AA genotype of IFN-γ +874 polymorphism, might be dependent on the population. 

**Table 1:** Sequences of used primers for interferon gamma genotyping

Primers	Sequences	Product Lengths	Ref.
For sequencing	**F**: 5'- TCGTTGCTCACTGGGATTTTG-3′	322 bp	(12)
	**R**: 5′-CATCTACTGTGCCTTCCTGT-3′		
For ARMS-PCR	**Common (R): ** 5'--3'	264 bp	(10)
	**Allele A (F):** 5'-		
	**Allele T (F):** 5'-		
Control in ARMS-PCR	**F**: 5'- TCGTTGCTCACTGGGATTTTG-3′	470 pb	(12)
	**R**: 5'-TCAACAAAGCTGATACTCCA-3'		(10)

**Table 2:** Characteristics of study subjects (n=140): healthy controls and chronic HBV patients

Characteristics	Controls (n=70)	Patients (n=70)
Age, mean ± SD	38.49 ± 11.22	41.05 ± 13.16
Gender		
Male	55	55
Female	15	15
Smoking status		
Yes	44	44
No	26	26
Alcohol Status		
Yes	2	2
No	67	67

**Table 3:** Genotype and allele frequencies of subject study (n = 140

	Genotypes			Allele Frequencies	
	AA	AT	TT	A	T
Control individuals (n = 70)	11 (16%)	41 (58%)	18 (26%)	0.45	0.55
Chronic HBV Patients (n = 70)	25 (36%)	30 (44%)	15 (21%)	0.57	0.43

**Table 4:** Association of IFN-γ +874 polymorphism with chronic HBV infection in different genetic models (n = 140

Model	Genotype	OR (95% CI)	P-value	AIC
Codominant	AA AT TT	1.00 3.18 (1.35-7.52) 2.78 (1.03 – 7.52)	0.021*	194.1

Dominant	AA AT&TT	1.00 3.05 (1.35 – 6.89)	0.0057*	192.2
Recessive	AA&AT TT	1 1.27 (0.58 – 2.81)	0.55	199.4
Overdominant	AA&TT AT	1.00 1.91 (0.97-3.76)	0.059	196.2
Log-additive	---	1.67 (1.02-2.73)	0.038*	195.5

AIC = Akaike information criterion,

* P < 0.05

Conversely, and with consistency with other studies (11, 21, 22), the AT and TT genotypes were significantly lower in the patients group compared with the control group (*P* < 0.05). These two genotypes together reduced the risk of chronic HBV with odd ratio of 0.33 (95% CI = 0.150 – 0.753). This might suggest that there is no difference in the presence of one copy or two copies of the allele T of INF-γ +874 polymorphism, in term of the susceptibility to chronic HBV infection.

Our results showed that the model (AA vs. AT&TT) was the best genetic model. This might support our conclusion that the absence of allele T increases the risk of chronic HBV in term of IFN-γ 874+ A/T polymorphism.

Different studies showed that IFN-γ 874+ A/T polymorphism is associated with an increased risk of chronicity of HBV in Caucasians but not in Asians. To our knowledge, our study is the first study that showed an association between the IFN-γ 874+ A/T polymorphism with chronic HBV in Arab countries. This association might support the suggested similarity of our population with Caucasian rather than Asian ethnic. 

In conclusion, the AA genotype of IFN-γ 874+ A/T polymorphism increased the risk of chronic HBV in the Syrian population. However, a small number of subjects studied remains a limiting factor. Therefore, these findings need to be further confirmed by conducting studies on a larger number of subjects. 
